# Structure Modification of an Active Azo-Compound as a Route to New Antimicrobial Compounds

**DOI:** 10.3390/molecules22060875

**Published:** 2017-05-25

**Authors:** Simona Concilio, Lucia Sessa, Anna Maria Petrone, Amalia Porta, Rosita Diana, Pio Iannelli, Stefano Piotto

**Affiliations:** 1 Department of Industrial Engineering, University of Salerno, Via Giovanni Paolo II 132, 84084 Fisciano, Italy; 2 Department of Pharmacy, University of Salerno, Via Giovanni Paolo II 132, 84084 Fisciano, Italy; lucsessa@unisa.it (L.S.); Anpetrone@unisa.it (A.M.P.); aporta@unisa.it (A.P.); piannelli@unisa.it (P.I.); piotto@unisa.it (S.P.); 3 PhD Program in Drug Discovery and Development, University of Salerno, Via Giovanni Paolo II 132, 84084 Fisciano, Italy; 4 Department of Chemical Science, University of Napoli “Federico II”, Cupa Nuova Cintia 21, 80126 Napoli, Italy; rosita.diana@libero.it

**Keywords:** azo-compound, antimicrobial, synthesis, QSAR

## Abstract

Some novel (phenyl-diazenyl)phenols **3a**–**g** were designed and synthesized to be evaluated for their antimicrobial activity. A previously synthesized molecule, active against bacteria and fungi, was used as lead for modifications and optimization of the structure, by introduction/removal or displacement of hydroxyl groups on the azobenzene rings. The aim of this work was to evaluate the consequent changes of the antimicrobial activity and to validate the hypothesis that, for these compounds, a plausible mechanism could involve an interaction with protein receptors, rather than an interaction with membrane. All newly synthesized compounds were analyzed by ^1^H-NMR, DSC thermal analysis and UV-Vis spectroscopy. The in vitro minimal inhibitory concentrations (MIC) of each compound was determined against Gram-positive and Gram-negative bacteria and *Candida albicans*. Compounds **3b** and **3g** showed the highest activity against *S. aureus* and *C. albicans*, with remarkable MIC values of 10 µg/mL and 3 µg/mL, respectively. Structure-activity relationship studies were capable to rationalize the effect of different substitutions on the phenyl ring of the azobenzene on antimicrobial activity.

## 1. Introduction

Antimicrobial therapy is a powerful tool for the treatment of several diseases, and is a keystone of modern medicinal practice. Infections by pathogenic microorganisms are of great concern in many fields, particularly in medical devices, hospital surfaces, surgery equipment, health care products, food packaging and storage. Infections are generally combated with antimicrobial agents [1], however, some bacteria, upon mutating their genes, have become resistant to common antibiotics and this makes difficult their elimination [2,3,4,5,6]. Consequently, the increased resistance of microorganisms to the currently used antimicrobials has led to the evaluation of other agents that might have antimicrobial activity [7,8].

Recently, our research group described the synthesis and properties of some azobenzene-based compounds and their antimicrobial properties both as single molecules [9] and dispersed in a polymer matrix [10,11,12]. In one of these works, we also selected the best candidates by preliminary in-silico test of ADMET properties and then we synthesized the azo compounds with lowest in-silico toxicity values according to the classic diazo coupling reaction scheme [9,13]. The majority of synthesized compounds exhibited high antibacterial activity against *S. aureus* and antifungal activity against *C. albicans*, but they were inactive against Gram-negative bacteria such as *Pseudomonas aeruginosa* and *Salmonella typhimurium*. Because of the structural similarity with stilbenes, these compounds can share a similar mechanism of action. They could inhibit ATP synthase binding at the interface between α and γ subunits [9].

Azobenzene-based derivatives possess interesting features that warrant further investigation. Among others, the presence of photo-inducible isomerism can be exploited in remodeling lipid membranes [14] or to modulate the membrane interaction of peptides [15]. Currently, we are evaluating the possibility to exploit azobenzene-based derivatives to synthesize tunable metal complexes [16,17] having antimicrobial activity.

In this work, we introduced some structure modifications to enhance the biological activity of the previously obtained azo-compounds. In particular, we aimed to evaluate the change in activity related to different types of substituents linked on the azobenzene structure.

**Figure 1:**
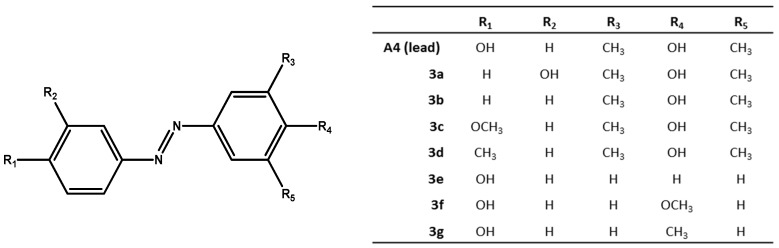
Substituted-(phenyldiazenyl)phenol derivatives **3a**–**g**.

We chose compound **A4** from [9], one the best antibacterial azo-compound we synthesized, as lead compound to synthesize several analogues (see Figure 1). We introduced simple molecular modifications on both azobenzene rings, in order to evaluate the role of the phenolic hydroxyl group in the mechanism of action. The substituents are joined to the fixed moiety azobenzene: the modifications involved moving of the phenolic hydroxyl group from the *para*- to the *meta*-position, or removal of the phenolic hydroxyl group, and replacing the phenolic hydroxyl group by methoxy and methyl groups, to study and compare their antimicrobial activity.

Quantitative Structure-Activity Relationships (QSAR) are mathematical frameworks that relate the molecular structures of compounds with their natural activities in a quantitative way [7]. The main success of the QSAR method is the possibility to estimate the properties of new chemical compounds without the need to synthesize and test them. One of the most efficient and popular technique is the Genetic Algorithm Search (GAS). GAS are inspired by natural evolution principles where variables play the role of genes (in this case a set of descriptors) in an individual of the species. A group of random individuals (population) evolves according to a fitness function (in this case the antimicrobial activity) that determines the persistence of the individuals. The algorithm exploits selection, mutation, and crossover operations on the genes. The QSAR correlates biological activity with structural and chemical properties. The results of the QSAR analysis permitted to develop a model of activity in which electronics transition are crucial. This approach will permit a more efficient design of azobenzene-based antibiotics.

## 2. Results and Discussion

### 2.1. Chemistry

The substituted-(phenyldiazenyl)phenols **3a**–**g** derivatives are reported in Figure 1.

**Scheme 1:**
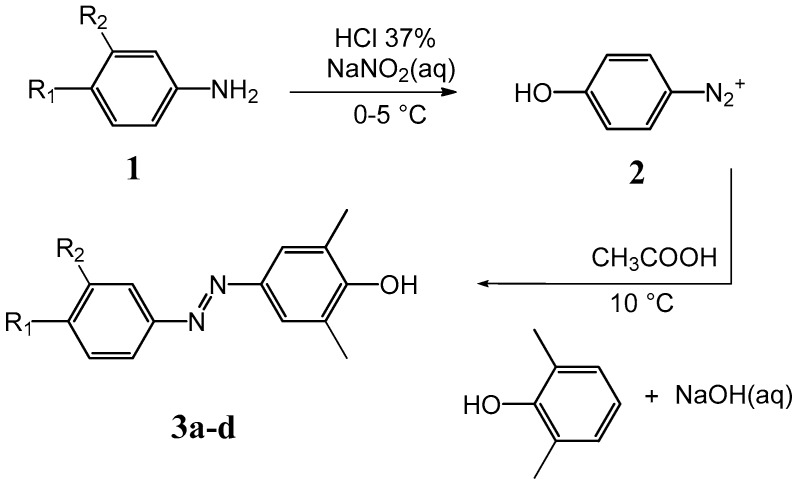
Synthetic route for compounds **3a**–**d**.

**Scheme 2:**
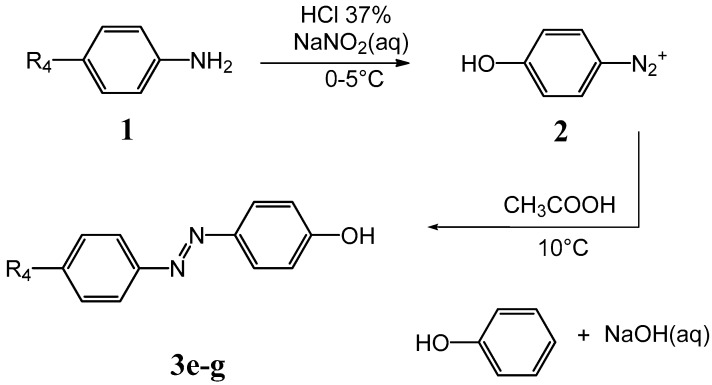
Synthetic route for compounds **3e**–**g**.

All derivatives **3a**–**g** were synthesized according to the methods outlined in Scheme 1 and Scheme 2.

Synthesis of compounds **3a**–**g** was accomplished according to the classic diazo coupling reaction scheme. An aromatic amine **1** (depending on the **A4** analogue) was suspended in an acid solution and cooled in an ice bath. Sodium nitrite was added, obtaining a suspension of the diazonium salt **2**. Separately, an aqueous solution of NaOH containing the appropriate phenol was prepared and the two solutions mixed under stirring, maintaining the pH values around 10 for 30 min. The final solution was acidified until a dark red precipitate of the azo compound was formed. ^1^H-NMR experiments were performed to confirm all structures.

### 2.2. Thermal and Optical Properties

In Table 1, the thermal and optical properties of the synthesized compounds are reported. Analogues **3a**, **3b**, **3d** and **3e** showed only a melting peak in the first heating run and they were not able to crystallize from the melt. In particular, **3a** melts at 114.4 °C, **3b** at 87.7 °C, **3d** at 89.3 °C and 162.0 °C. Compound **3c** showed a sharp melting peak in the first heating run at 128.3 °C and a crystallization peak at 94.6 °C in the cooling run. When heated in the second run, it showed the same melting peak as in the first run. Compound **3f** showed a sharp melting peak in the first heating run at 147.3 °C and a crystallization peak at 110.6 °C in the cooling run; when heated in the second run, it showed the same melting peak as in the first run. **3g** showed a sharp melting peak in the first heating run at 158.7 °C and a crystallization peak at 90.7 °C in the cooling run. When heated in the second run, it showed the same melting peak as in the first run.

The spectral region 650–240 nm was investigated by UV-Vis spectrophotometry, at a concentration of about 3.0 × 10^−5^ mol L^−1^ of azo-compound in acetonitrile solution (Table 1). The UV-visible spectra for **3a**–**g** are qualitatively only depend on the azobenzene unit, which is the same for all compounds. The UV absorption spectra of **A4** analogues in *trans* configuration, showed the typical absorption bands of the electronic transitions of the azobenzene chromophore [18].

### 2.3. Antimicrobial Activity

The MICs of the synthesized analogues and of lead compound **A4** were determined by the microbroth dilution for *S. aureus* A170, *P. aeruginosa* ATCC-27853 strains and *C. albicans* SC5314 (Table 2). 

**Figure 2:**
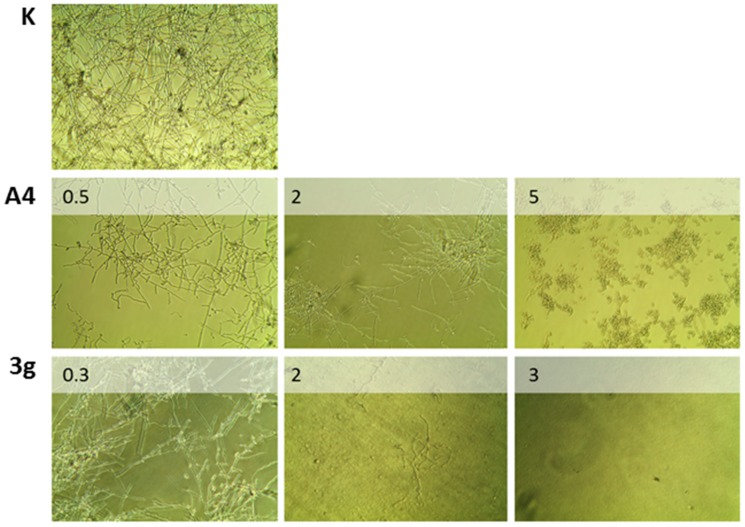
Inhibition of hyphae formation in *Candida albicans* at different concentrations (μg/mL) of **A4** and **3g** (20× optical zoom).

Analogues **3b** and **3g** are the most active antimicrobial analogue, exhibiting activity at a concentration lower than the lead compound concentration. In particular, **3b** is the only analogue able to inhibit the growth of 100% of *S. aureus* at a concentration of 10 µg/mL; indeed for other compounds only the MIC_50_ values are reported (Table 2). Compound **3g** is the most active antifungal analogue, showing an MIC_0_, which means 100% of inhibition of *C. albicans* duplication already at 3 µg/mL (see also Figure 2).

Compared to the reference molecule, other analogues such as **3a**, **3e** and **3f** show lower activities. Nevertheless, they still possess antimicrobial and antifungal activity, even if at concentrations higher than **A4**. Compounds **3c** and **3d** lose the antibacterial and antifungal activity and none of the analogues exhibit antimicrobial activity against *P. aeruginosa,* as already observed for previously synthesized similar compounds [9].

Interestingly, all the compounds show antibacterial activity only against Gram-positive bacteria. This can be correlated with the different composition and structure of the Gram-negative bacteria. Figure 2 shows the total inhibition of germination and hyphae formation of azo compound **3g** on *C. albicans*, at 3 μg/mL. In the absence of azo compound, an extensive hyphae formation was observed, whereas when **3g** was present, hyphae formation was severely hampered in a concentration-dependent manner.

### 2.4. QSAR Models

QSAR models were performed to predict the activity of azobenzene molecules against *S. aureus*. We have generated several QSAR models using different training and test sets and we have retained only statistically significant models. The efficacy of the molecules are estimated as the inverse of MIC_50_. Therefore, higher efficacy corresponds to higher values of 1/MIC_50_. After careful evaluation of all models, we have chosen the following:

(1) $$\frac{1}{{\mathrm{MIC}}_{50}}=0.0789\cdot \mathrm{VR}1_{\mathrm{Dzp}}-0.0580\cdot \mathrm{SpMin}8_{Bhi}+0.116$$

where VR1_Dzp is the Randic-like eigenvector-based index from Barysz matrix/weighted by polarizabilities and standardized by range, and SpMin8_Bhi is the smallest absolute eigenvalue of Burden modified matrix—n 8/weighted by relative first ionization potential standardized by range.

These descriptors belong to the Barysz distance matrix-based descriptors, and to the Burden modified matrix. The best model correlates polarizabilities and ionization potential with the biocidal activity and, therefore, suggests an interaction with a protein receptor rather than with the bacterial cell membrane. The comparison between the experimental biocidal activity against *S. aureus* and the predicted MIC_50_ calculated with Equation (1) is shown in Table 3.

The validation tests used to check the predictive ability of the model are listed in Table 4.

The validation tests indicate good predictive capability of the model, but there are a few molecules showing a large error. In particular, molecule **3d** displays the highest error. This could be associated to the low solubility of **3d** that, in fact, has the largest value of AlogP. AlogP is a thermodynamic descriptor and represents hydrophobicity [19].

## 3. Materials and Methods 

### 3.1. General Information

All the reagents and solvents were purchased from Sigma-Aldrich (Milan, Italy) and used without further purification. Optical observations were performed by using a Jenapol microscope (Zeiss S.p.A., Milano, Italy) fitted with a THMS 600 hot stage (Linkam, Waterfield, Epsom, Tadworth, UK). Phase transition temperatures and enthalpies were measured using a Pyris 1 DSC scanning calorimeter (PerkinElmer, Waltham, MA, USA) at a scanning rate of 10 °C/min, under nitrogen flow. UV-Vis absorption spectra of the samples were recorded at 25 °C in acetonitrile solution, on a Perkin Elmer Lambda 19 spectrophotometer. The spectral region 650−240 nm was investigated by using cell path length of 1.0 cm. Azobenzene chromophore concentration of about 3.0 × 10^−5^ mol L^−1^ was used. ^1^H-NMR spectra were recorded with a DRX/400 spectrometer (Bruker, Billerica, MA, USA). Chemical shifts are reported relative to the residual solvent peak (dimethylsulfoxide-*d*_6_: H = 2.50 ppm). HPLC runs were carried out on a C_18_ column (Jupiter, 5 μm, 300 Å, LC column 150 × 4.60 mm, Phenomenex, Torrance, CA, USA) at a flow rate of 1.0 mL/min. The gradient [solution A: TFA (0.1%); solution B: TFA (0.07%), H_2_O (5%), CH_3_CN (95%)] was 20−70% B over 20 min. HPLC analysis was performed on an 1260 Infinity Series system (Agilent, Santa Clara, CA, USA) with UV detection at 280, 320, 350 and 380 nm.

### 3.2. General Method of the Synthesis of (Phenyl-Diazenyl)Phenols Derivatives ***3a**–**d***

A suspension of the proper aromatic amine (0.0183 mol) in a solution containing water (38 mL) and HCl 37% (*w/w*, 4 mL) was cooled at 0−5 °C in a water-ice bath. A solution of sodium nitrite (1.39 g, 0.0202 mol) dissolved in water (8.0 mL) was added dropwise, giving a suspension of the diazonium salt (solution A). Separately, a solution containing NaOH (0.7 g, 0.0175 mol) in water (50 mL) with 2,6-dimethylphenol (2.23 g, 0.0183 mol) was prepared (solution B). Solution A was added dropwise to solution B, under stirring at 12 °C. The system was left reacting for 30 min, maintaining the pH = 10−11. Then the final solution was slowly added to 103 mL of an acid solution (100 mL of water and 3 mL of acetic acid), and then stirred for 30 min at 15 °C. A dark red precipitate of the azo compound was formed. The crude precipitate was filtered and dried under vacuum. Yields ranged between 30% and 40%.

*3’*-*Hydroxy*-(*4*-*hydroxy*-*3*,*5*-*dimethyl*)-*azobenzene* (**3a**): 3-Aminophenol and 2,6-dimethylphenol were used as starting reagents. The crude product was extracted and crystallized from boiling *n*-octane (100 mL) and dried. Final crystallization from boiling water/ethanol (3:1) gave pure **3a** as orange crystals. Polarized optical microscopy showed a needle-like crystalline form. Yield: 30%. ^1^H-NMR (DMSO-*d*_6_): δ (ppm) = 7.55 (s, 2H), 7.34 (m, 2H), 7.19 (d, 1H), 6.90 (d, 1H), 2.27 (s, 6H). HPLC chromatogram of **3a** shows a single peak, ensuring a purity >95% (Supplementary Materials Figure S1).

*4*-*Hydroxy*-*3*,*5*-*dimethylazobenzene* (**3b**): Aniline and 2,6-dimethylphenol were used as starting reagents. The crude product was extracted and crystallized from boiling water/ethanol (3:1) and dried, to give pure **3b** as amber crystals. Polarized optical microscopy showed a needle crystalline habitus. Yield: 35%.^1^H-NMR (DMSO-*d*_6_): δ (ppm) = 7.82 (d, 2H), 7.55 (m, 5H), 2.27 (s, 6H). HPLC chromatogram of **3b** shows a single peak, ensuring a purity >95% (Supplementary Materials Figure S2).

*4*’-*Methoxy*-(*4*-*hydroxy*-*3,5*-*dimethyl*)*azobenzene* (**3c**): *p*-Anisidine and 2,6-dimethylphenol were used as starting reagents. The extraction and crystallization of the crude product from boiling water/ethanol (3:1) gave pure **3c** as yellow crystals. Polarized optical microscopy showed a needle-like crystalline form. Yield: 40%. ^1^H-NMR (DMSO-*d*_6_): δ (ppm) = 7.81 (d, 2H), 7.51 (s, 2H), 7.11 (d, 2H), 3.85 (s, 3H), 2.26 (s, 6H). HPLC chromatogram of **3c** shows a single peak, ensuring a purity >95% (Supplementary Materials Figure S3).

*4*’-*Methyl*-(*4*-*hydroxy*-*3*,*5*-*dimethyl*)*azobenzene* (**3d**): *p*-Toluidine and 2,6-dimethylphenol were used as starting reagents. The extraction and crystallization of the crude product from boiling water/ethanol (3:1) gave pure **3d** as orange crystals. Polarized optical microscopy showed a plate-like crystalline form. Yield: 38%. ^1^H-NMR (DMSO-*d*_6_): δ (ppm) = 7.72 (d, 2H), 7.54 (s, 2H), 7.36 (d, 2H), 2.39 (s, 3H), 2.27 (s, 6H). HPLC chromatogram of **3d** shows a single peak, ensuring a purity >95% (Supplementary Materials Figure S4).

### 3.3. General Method of the Synthesis of (Phenyl-Diazenyl)Phenols Derivatives ***3e**–**g***

A suspension of the proper aromatic amine (0.0183 mol) in a solution containing water (38 mL) and HCl 37% (*w/w*, 4 mL) was cooled at 0−5 °C in a water-ice bath. A solution of sodium nitrite (1.39 g, 0.0202 mol) dissolved in water (8 mL) was added dropwise, giving a suspension of the diazonium salt (solution A). Separately, a solution containing NaOH (0.7 g, 0.0175 mol) in water (100 mL, pH = 14) with phenol (1.72 g, 0.0183 mol) was prepared (solution B). Solution A was added dropwise to solution B, under stirring at 12 °C. The system was left reacting for 30 min, maintaining the pH = 11. The final solution was slowly added to 300 mL of an aqueous solution of acetic acid (pH = 5) and then stirred for 30 min at 15 °C. In this step, to facilitate the precipitation of the azo compound I sodium acetate powder (0.5 g) was added and the mixture stirred for 20 min in water-ice bath. A dark precipitate of the azo compound was obtained, filtered and dried under vacuum. Yields ranged between 50% and 60%.

*4*’-*Hydroxyazobenzene* (**3e**): Aniline and phenol were used as starting reagents. The crude product was extracted and crystallized from boiling *n*-octane (100 mL) and dried. Final crystallization from boiling water gave pure **3e** as orange/yellow crystals. Polarized optical microscopy allowed the observation of needle-like crystals of the azo compound. Yield: 56%. ^1^H-NMR (DMSO-*d*_6_): δ (ppm) = 7.81 (t, 4H), 7.54 (m, 3H), 6.96 (d, 2H). HPLC chromatogram of **3e** shows a single peak, ensuring a purity >95% (Supplementary Materials Figure S5).

*4*’-*Hydroxy*-*4*-*methoxyazobenzene* (**3f**): *p*-Anisidine and phenol were used as starting reagents. The crude product was extracted and crystallized from boiling *n*-octane (100 mL) and dried. Final crystallization from boiling water/ethanol (3:1) gave pure **3f** as dark orange crystals. Polarized optical microscopy showed a plate-like crystalline form. Yield: 58%. ^1^H-NMR (DMSO-*d*_6_): δ (ppm) = 7.80 (dd, 4H), 7.51 (s, 2H), 7.11 (d, 2H), 6.93 (d, 2H), 3.86 (s, 3H). HPLC chromatogram of **3f** shows a single peak, ensuring a purity >95% (Supplementary Materials Figure S6).

*4*’-*Hydroxy*-*4*-*methylazobenzene* (**3g**): *p*-Toluidine and phenol were used as starting reagents. The crude product was extracted and crystallized from boiling *n*-octane (100 mL) and dried. Final crystallization from boiling water/ethanol (3:1) gave pure **3g** as brilliant yellow crystals. Polarized optical microscopy showed a needle-like crystalline form. Yield: 60%. ^1^H-NMR (DMSO-*d*_6_): δ (ppm) = 7.75 (dd, 4H), 7.54 (s, 2H), 7.35 (d, 2H), 6.93 (d, 2H), 2.39 (s, 3H). HPLC chromatogram of **3g** shows a single peak, ensuring a purity >95% (Supplementary Materials Figure S7).

### 3.4. Antimicrobial Tests

#### 3.4.1. Bacterial Strains and Minimum Inhibitory Concentrations 

The in vitro minimal inhibitory concentrations (MIC) of each compound was determined against *Candida albicans* SC5314 by the micro-broth dilution method in 96-well plates according to the guidelines suggested by the Clinical and Laboratory Standards Institute (CLSI) [20] using three separate plates each containing the same batch of azo compounds.

Microtiter plates containing 100 μL of two-fold serial dilutions of azo compounds in RPMI 1640 medium were inoculated with 100 μL of cells containing 2.5 × 10^3^ yeast/mL and incubated at 35 °C for 24 h. The resulting MICs were visually read as the lowest concentration of compound causing a reduction or an absence of growth (optically clear) in comparison to the drug-free growth control.

For *S. aureus* A170 a clinically isolated gentamicin resistant strain (kindly provided by Prof. R. Capparelli from the University of Naples, Naples, Italy) and *P. aeruginosa* ATCC-27853, MIC values of each compound were determined by the serial broth microdilution method as reported by Patton [21]. Therefore, flat-bottom polystyrene microtiter plates containing 100 µL of two-fold serial dilutions (six replicates per dilution) of azo compounds were inoculated with 100 µL of ~5 × 10^5^ CFU/mL of each bacterium grown in Mueller–Hinton broth 2. The controls were the wells contained broth only (negative control) and bacteria and broth (positive control).

Plates were incubated at 37 °C with shaking at 160 rpm for 24 h. Data were analyzed according to Patton et al. [21]. The optical density (OD) was determined just before the incubation (T_0_) and again after 24 h incubation (T_24_) at 600 nm. The OD for each replicate at T_0_ was subtracted from the OD for each replicate at T_24_. The adjusted OD of each control well was then assigned a value of 100% growth. The MIC is reported as the lowest concentration of azo compounds, which results in 100% or 50% inhibition of growth.

#### 3.4.2. *Candida Albicans* Morphological Analysis

Hyphal growth of *Candida*-treated cells was induced using RPMI 1640 medium. Stationary yeast cells were inoculated into a fresh pre-warmed medium at a density of 6 × 10^6^ cells/mL in a flat-bottom 96 well microtiter plates. Different concentrations of azo compounds (ranging from 1 to 50 μg/mL), were added to each well. After incubation at 37 °C for 24 h, each microtiter plate was examined using an inverted microscope to monitor phenotypic modification and hyphae formation. 

### 3.5. QSAR Analysis

For the series of 14 azobenzenes molecules we have calculated 3780 chemical descriptors by means of the PaDel tool [22] and the Genetic algorithms available in the program Materials Studio [23]. The 3780 parameters calculated have been standardized by range between −1 and 1. The regression was made using the inverse of MIC_50_ against *S. aureus*. 

The number of descriptors in the regression equation was set to 2, and Population and Generation were set to 5000 and 50,000, respectively. Mutation probability was 0.1, and the smoothing parameter was 0.5. The choice of keeping the number of descriptors low was intended to avoid overfitting and to determine the most important molecular descriptors only, whereas all the other parameters have been chosen as recommended by the software developers. In all simulations, the algorithm reached convergence within 270 and 380 generations.

The choice of the models was based on Friedman’s Lack of Fit (LOF) calculated as follows:

(2) $$LOF=\frac{SSE}{M{\left[1-\lambda \left(\frac{c\;+\;dp}{M}\right)\right]}^{2}}$$

where *SSE* is the sum of squares of errors, *c* is the number of terms in the model, other than the constant term, *d* is a scaled smoothing parameter, *p* is the total number of descriptors contained in all model terms (again ignoring the constant term), *M* is the number of samples in the training set, and *λ* is a safety factor, with a value of 0.99, to ensure that the denominator of the expression can never become zero, and so the LOF is always well-defined.

An adjusted R^2^ estimation, where the variance is reduced in proportion to the size of the estimated model. The adjusted R^2^ is calculated as follows:

(3) $$1-\frac{SSE/\left(n-p\right)}{SST/\left(n-1\right)}$$

where *p* is the number of parameters in the regression equation. *SST* is the total sum of squares, and *n* is the number of data points from which the model is built. Compared to R^2^, this measure penalizes large equations. The cross validated R-squared (R^2^(CV)) value is also a key measure of the predictive power of a model. The closer the value is to 1.0, the better the predictive power. For a good model, R^2^(CV) should be fairly close to R^2^. If R^2^(CV) is much less than R^2^, the model equation is probably overfitting the data. A model with an R^2^(CV) value of 0.0 or less has no predictive power at all, according to the cross-validation criterion. The R^2^(CV) is calculated as follows:

(4) $$1-\frac{PRESS}{SST}$$

where *PRESS* is the predictive sum of squares of a model. After a required group of data is deleted, the remaining data in *x* is used to produce a new model for *y*. These predicted values of *y* are compared with the exact values that have been excluded:

(5) $$PRESS\;\left(n\right)=\;\overset{n}{\underset{i=0}{\sum }}{\left(y_{i}-{y^{\prime }}_{i}\right)}^{2}$$

A model is produced for each row or group of rows excluded. An indication of whether or not the regression is statistically significant: Yes if F > Fcr, No if not. F is the significance-of-regression *F* value and Fcr is the critical SOR *F* value (95%).

The F test is a standard statistical test for the equality of the variances of two populations having normal distributions. Here, it is used to test whether the variance in the data which is explained by the regression is much larger than the variance remaining due to errors. If this is the case, then the model is said to be significant, rather than one which simply fits the noise [24]. The significance-of-regression *F* value is defined as:

(6) $$\frac{SSR/\left(p-1\right)}{SSE/\left(n-p\right)}$$

This parameter is used to determine whether or not the regression is statistically significant. The critical significance-of-regression (SOR) *F* value is the critical point of the *F* distribution of degrees *n* − *p* and *p* − 1 evaluated for probability 0.05 (at 95% confidence level). In conjunction with the significance-of-regression *F* value, it is used to determine whether or not the regression is significant.

## 4. Conclusions 

Some novel derivatives of (4’-hydroxy-(4-hydroxy-3,5-dimethyl)azobenzene) were designed, synthesized, and biologically evaluated as antimicrobial agents. The majority of the synthesized compounds exhibited significant antibacterial activity against *S. aureus* and antifungal activity against *C. albicans*, but they were inactive against Gram-negative bacteria such as *P. aeruginosa*. The best results were obtained for 4-hydroxy-3,5-dimethylazobenzene (**3b**), which showed higher antibacterial activity against *S. aureus* than lead compound. The best antifungal activity was obtained for 4’-hydroxy-4-methylazobenzene (**3g**) that was able to inhibit the growth of 100% of *C. albicans* at a concentration five times lower than the lead compound.

Structure-activity relationship studies could rationalize the effect of different substitution patterns on the phenyl ring of the azobenzene on antimicrobial activity. Changing the electronic nature and the position of the substituent group attached to the aromatic ring led to changes in the observed MIC. These observations suggest that the killing ability depends mainly by the interaction with a not yet identified receptor. The best QSAR model explains the role of polarity and ionizability in determining the killing capacity of the synthetized compounds and suggests that these molecules interact with protein receptors and that the interaction with membranes is of minor importance. The solubility and the partition coefficient may rationalize the apparent poor activity of the compound **3d**. Taken together, these results can be extremely promising for the design of novel azobenzene-based antibiotics.

## Figures and Tables

**Table 1:** Thermal and optical properties of compounds **3a**–**g**.

Molecule	Thermal Characterization				Optical Characterization	
	T_m_ (°C)	∆H_m_ (J/g)	T_c_ (°C)	∆H_c_ (J/g)	λ_max_ (nm)	ε_max_ (L mol^−1^ cm^−1^)
**3a**	114.4	5.7	-	-	358	9300
**3b**	87.7	78.0	-	-	344	18,300
**3c**	128.3	98.1	94.6	92.1	360	14,000
**3d**	89.3	86.1	-	-	354	11,400
**3e**	162.0	149.8	-	-	344	14,470
**3f**	147.3	123.3	110.6	104.5	355	14,200
**3g**	158.7	139.4	90.7	83.7	358	14,350

T_m_ = melting temperature, from DSC analysis, 10 °C/min, nitrogen flow; T_c_ = crystallization temperature, from DSC cooling run; Instrument error ±0.5 °C. ∆H_m_/∆H_c_ = melting/crystallization enthalpy, evaluated by integration of the peak. Experimental error ±5%. λ_max_ = wavelength at the principal absorption maximum, ε_max_ = molar extinction coefficient at absorption maximum.

**Table 2:** Antimicrobial and antifungal activity of **A4** analogues. Geometric mean MICs (µg/mL) after 24 h.

Molecule	*S. aureus*	*P. aeruginosa*	*C. albicans*
	MIC_50_		MIC_2_
**A4 (lead)**	15 (MIC_100_ 20 *)	>35	15 (MIC_0_ 17 *)
**3a**	30	>35	25
**3b**	7 (MIC_100_ 10)	>35	10
**3c**	>35	>35	>35
**3d**	>35	>35	>35
**3e**	25	>35	20
**3f**	25	>35	25
**3g**	7 (MIC_100_ 20)	>35	MIC_0_ 3

MIC_50_: Minimum Inhibitory Concentration required to inhibit the growth of 50% of bacteria. MIC_100_: Minimum Inhibitory Concentration required to inhibit the growth of 100% of bacteria. MIC_2_: Minimum Inhibitory Concentration required to inhibit the growth of 50% of fungus. MIC_0_: Minimum Inhibitory Concentration required to inhibit the growth of 100% of fungus. The values are the geometric mean of at least three determinations. * Data from ref. [9].

**Table 3:** Comparison of experimental and calculated antimicrobial activity against *S. aureus*.

Molecule	MIC_50_ Experimental (μg/mL)	Actual Values for C:1/MIC_50_	ALogP	Equation (1): Predicted Values	Equation (1): Residual Values	MIC_50_ Predicted
**A4**	15	0.067	2.8	0.0513	0.0154	19
**3a**	30	0.033	2.8	0.0520	−0.0187	19
**3b**	7	0.143	5.0	0.1460	−0.0030	7
**3c**	80 *	0.013	3.0	0.0214	−0.0089	50
**3d**	80 *	0.013	7.2	0.0357	−0.0232	30
**3e**	25	0.040	1.8	0.0452	−0.0052	22
**3f**	25	0.040	0.7	0.0909	−0.0509	11
**3g**	7	0.143	3.2	0.0950	0.0479	10
**A1**	17	0.059	0.8	0.0756	−0.0168	13
**A2**	15	0.067	2.6	0.0822	0.0155	12
**A3**	14	0.071	0.2	0.0699	0.0015	14
**A5**	18	0.056	4.3	0.0625	−0.0069	16
**B10**	80 *	0.013	0.3	0.0142	−0.0017	70
**B11**	80 *	0.013	1.1	0.0142	−0.0017	70

* Molecules that did not show activity for concentration larger than 35 µg/mL, have been arbitrarily assigned to 80 to perform the GFA.

**Table 4:** Validation of the genetic function results.

Parameters	Value
Friedman LOF	0.00302
R-squared	0.802
Adjusted R-squared	0.766
Cross validated R-squared	0.654
Significant Regression	Yes
Significance-of-regression F-value	22.321
Critical SOR F-value (95%)	5.2341
Replicate points	0
Computed experimental error	0.000
Number or points for Lack-of-fit test	11
Min expt. error for non-significant LOF (95%)	0.0201

## Supplementary Materials

The Supplementary Materials are available online.
