# Supplementary material for: Structure Modification of an Active Azo-Compound as a Route to New Antimicrobial Compounds
Source: Molecules. 2017 May 25;22(6):875. doi: 10.3390/molecules22060875 (PMC6152751; doi:10.3390/molecules22060875)
Supplement: Supplementary file 1 [file molecules-22-00875-s001.pdf]

Supplementary Materials

Structure modification of an active azo-compound as a route to new antimicrobial compounds

Simona Concilio[a,*], Lucia Sessa[b], Anna Maria Petrone[b,c] Amalia Porta[b], Rosita Diana[d], Pio Iannelli[b] and Stefano Piotto[b]

[a] Department of Industrial Engineering, University of Salerno, Via Giovanni Paolo II 132, 84084 Fisciano (SA), Italy
[b] Department of Pharmacy, University of Salerno, Via Giovanni Paolo II 132, 84084 Fisciano (SA), Italy
[c] PhD Program in Drug Discovery and Development, University of Salerno, Via Giovanni Paolo II 132, 84084 Fisciano (SA), Italy
[d] Department of Chemical Science, University of Napoli "Federico II", Cupa Nuova Cintia, 21 - 80126 – Napoli, Italy
[*] Correspondence: sconcilio@unisa.it; Tel.: +39-089-964115

RP HPLC Chormatograms of compouds **3a**-**3g**, detected at 350 nm.

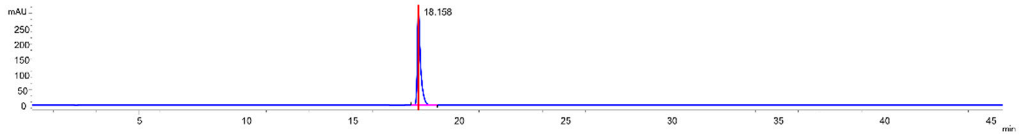

Figure S1 – Chromatogram of 3a

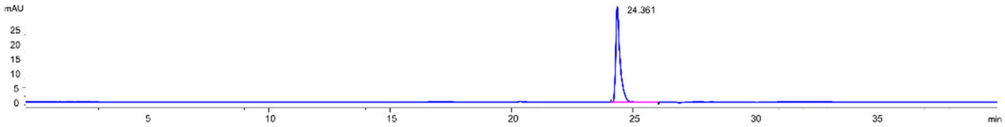

Figure S2 – Chromatogram of 3b

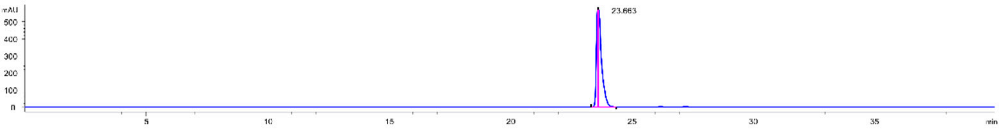

Figure S3 – Chromatogram of 3c

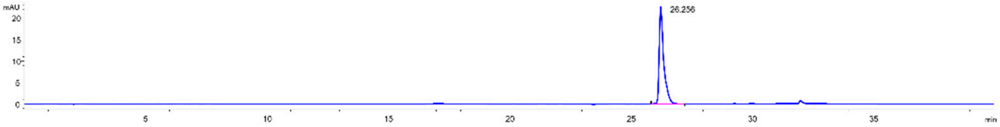

Figure S4 – Chromatogram of 3d

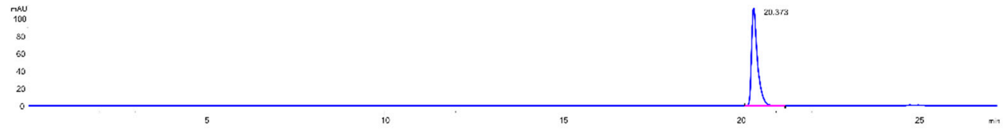

Figure S5 – Chromatogram of 3e

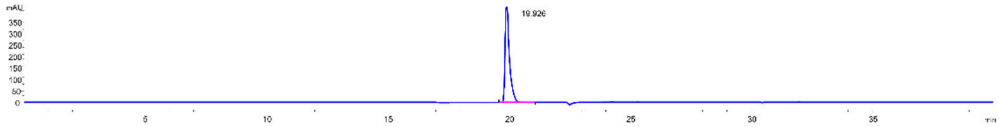

Figure S6 – Chromatogram of 3f

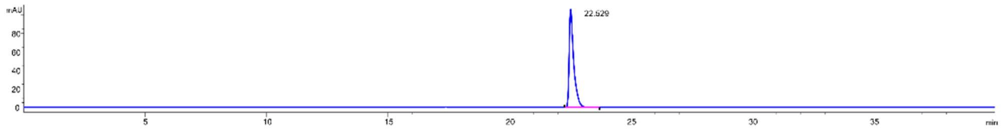

Figure S7 – Chromatogram of 3g
